# Rheology Measurements for Online Monitoring of Solids in Activated Sludge Reactors of Municipal Wastewater Treatment Plant

**DOI:** 10.1155/2014/590961

**Published:** 2014-01-14

**Authors:** Luciano Piani, Claudia Bruna Rizzardini, Adriano Papo, Daniele Goi

**Affiliations:** Dipartimento di Chimica, Fisica e Ambiente, Università degli Studi di Udine, Via Cotonificio, 108, 33100 Udine, Italy

## Abstract

Rheological behaviour of recycled sludge from a secondary clarifier of a municipal wastewater treatment plant was studied by using the rate controlled coaxial cylinder viscometer Rotovisko-Haake 20, system M5-osc., measuring device NV. The tests (hysteresis cycles) were performed under continuous flow conditions and following an ad hoc measurement protocol. Sludge shear stress versus shear rate curves were fitted very satisfactorily by rheological models. An experimental equation correlating the solid concentration of sludge to relative viscosity and fitting satisfactorily flow curves at different Total Suspended Solids (TTS%) was obtained. Application of the empirical correlation should allow the monitoring of the proper functioning of a wastewater treatment plant measuring viscosity of sludge.

## 1. Introduction

Biological activated sludge process is known as the most widely used one among wastewater treatments. This process has commonly good performance when a good solid-liquid separation between sludge and treated water is provided; nevertheless the sedimentation in the secondary clarifier depends on both the biological and physicochemical properties of the sludge [1, 2]. One of the most important features bound to the efficiency of the whole activated sludge process is to measure some sludge characteristics that can be used as tool to investigate the correct performance of treatment plants. Study of the sludge flow properties using rheology can be very useful in the process control and management [3].

Rheology could give valuable information when the design for a pumping scheme is requested [4] or it is necessary to recover transporting, storing, and spreading of operational parameters.

The procedures of sludge conditioning and dewatering have been studied by many researchers [5–8] and rheological characteristics of sludge have been effectively employed upgrading the sludge treatment process [9].

As well it is known rheological measurements may be used as an operational guide or control technique [10] and also to determine the relevancy of rheological parameters as indicators of sludge quality [11]; in particular, hysteresis cycles area was carried out online in aeration tanks of activated sludge as a way of detecting the appearance of filamentous bulkings and their evolution [12].

Experimental results obtained by testing sewage sludge taken from different municipal wastewater treatment works confirmed that solids concentration is the main parameter affecting sludge rheological behaviour [4].

Exponential and power laws were found as relationships connecting the most common rheological parameters such as yield stress or viscosity and TSS content [13, 14]; instead in 2011 Garakani connected viscosity with shear rate, temperature, and sludge concentration defined as Mixed Liquor Suspended Solids (MLSS) [15].

The aim of the present work is to study the rheological behaviour of a sludge sampled from the clarifier of a municipal wastewater treatment plant (WWTP) in which TSS and sedimentation properties remain significantly constant during the time, developing a standardized operative measurement procedure to obtain comparable and reproducible results. In this condition a link between rheological properties (relative viscosity) of activated sludge and its solid concentration (TSS%) by means of a mathematical model of *η*_*r*_ = *f* (TSS%, $\dot{\gamma }$) type was proposed.

A mathematical model of this kind can be a useful tool in monitoring the proper functioning of treatment plant in order to prevent critical settlement conditions by controlling solid concentration directly by measuring viscosity of sludge.

## 2. Experimental Methodology

### 2.1. Materials

Activated sludge was sampled in a municipal activated sludge wastewater treatment plant (Udine, Italy) from a secondary clarifier (recycled sludge). The WWTP of Udine consists of a traditional pretreatment, a primary treatment in which main solid particulate is removed by chemical coagulants addition and a secondary activated sludge system with final clarifier. Its potentiality is about 200000 equivalent inhabitants and a typical urban wastewater is treated in the WWTP.

The sampling was carried out monthly from January to September 2011.

The initial solid concentration of each sample was adjusted to achieve a number of samples having different solid concentrations by using the following procedures:

- thickening (by gravity) the original sample;
- diluting the thickened sludge with amounts of the same sludge liquid supernatant; the dilutions (thickened sludge parts per liquor parts in volume) were the following: 4 : 1, 3 : 1, 2 : 1, and 1 : 1 (range of dilution from 20% to 50% of thickened sludge).

Therefore from the original sludge sampled, the thickened sludge and a set of 4 other suspensions at different solid content are obtained.

In this work we take into consideration 3 sludge samples collected in different seasons of the year as representative (flow curves are very similar to the others) of all collected samples:

S1 (February), S2 (April), and S3 (July).

Characteristics of the sampled sludge mentioned above are reported in Table 1.

### 2.2. Apparatus and Procedures

Rheological tests were carried out in continuous flow conditions by using the rate controlled coaxial cylinder viscometer Rotovisko-Haake 20, system M5-osc., measuring device NV. According to theoretical considerations, in rotational rheometers the sensor NV is particularly adapted to rheological measurements in a low viscosity range corresponding to activated sludge viscosity, which is very close and slightly higher than water's.

The sensor system NV consists of the cup and a bell-shaped rotor. It is classified as a coaxial cylinder sensor system with two gaps for shearing the samples on the inside (inner gap) and on the outside (outer gap) of the rotor. In particular, supplying with the NV sensor system, the instrument usually presenting a classical concentric cylinders (CC) geometry becomes a rotational double coaxial cylinder viscosimeter with a double concentric cylinders (DCC) smooth geometry: this configuration consists of a stator inner radius of 17.5 mm, rotor inner radius of 17.85 mm, rotor outer radius of 20.1 mm, stator outer radius of 20.5 mm, measuring gaps of 0.35 mm (inner gap) and 0.40 mm (outer gap), and immersed height of the NV sensor of 60.0 mm.

The sludge sample volume used for each measurement was 9 mL. Temperature was kept strictly fixed at 20 ± 0.5°C. The choice of 20°C was established because it generally represents the mean temperature of the recycled sludge during the year. Before rheological measurements the absence of coarse particles was revealed sieving sludge samples.

Measurement conditions are summarized in the following procedures (hysteresis cycle):

- a preshear for one minute at constant shear rate of 100 s^−1^;
- increase of shear rate from 0 to 240 s^−1^ in 0.5 min (8 s^−2^ of constant shear acceleration);
- decrease of shear rate from 240 to 0 s^−1^ in 0.5 min.

A preshear is needed before carrying out rheological measurements:

- to have the same rheological history for every sample;
- to prevent sludge settling in the measuring cell;
- to avoid flow obstructions in the measuring cell during the first ramp of shear rate imposed that may occur when size of the internal structures of the sludge flocs is comparable to the size of the measuring gaps [16].

Rheological measurements were accomplished up to a maximum shear rate of 240 s^−1^ to ensure laminar flow conditions [16].

## 3. Results and Discussion

### 3.1. Continuous Flow Tests

**Figure 1:**
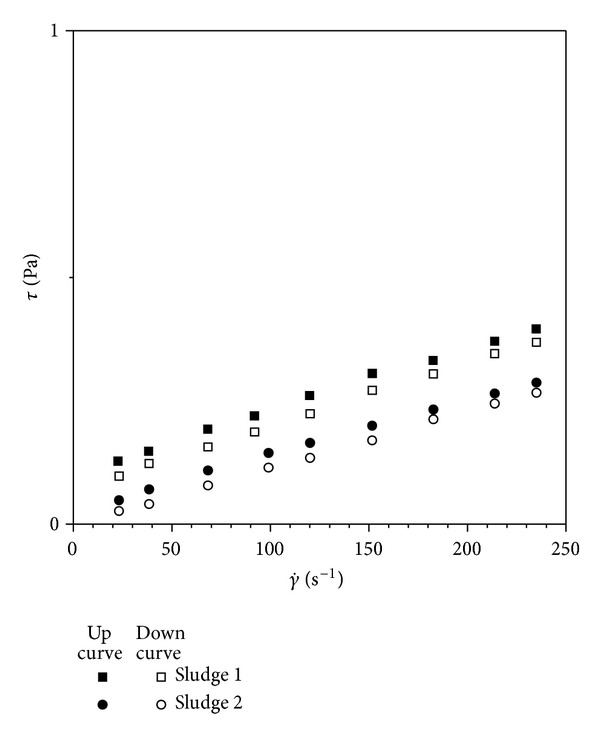
Hysteresis cycles drawn for some of the activated sludge investigated.

According to procedures mentioned above hysteresis cycles were carried out for all sludge sampled together with relative thickened sludge and diluted suspensions. The hysteresis loop presents both up and down curves of shear-thinning type and the rheological behaviour is slightly thixotropic (Figure 1).

**Figure 2:**
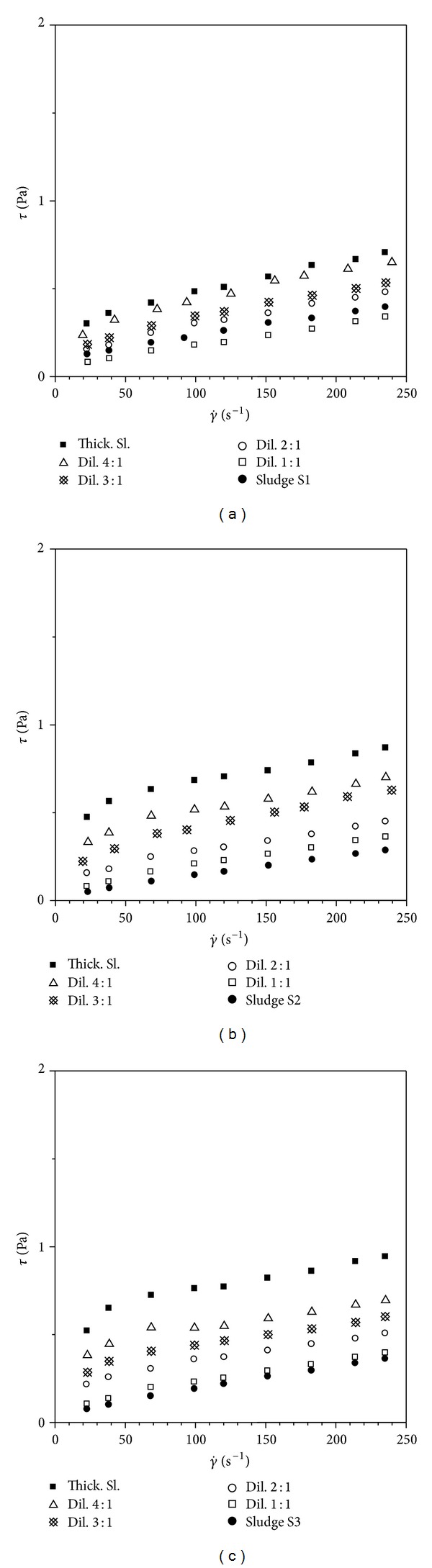
(a, b, c) Shear stress (*τ*) versus shear rate ($\dot{\gamma }$) flow curves determined for the activated sludges investigated.

The shear stress versus shear rate (Figures 2(a), 2(b), and 2(c)) and relative viscosity versus shear rate flow curves obtained for all the suspensions investigated in the present work (Table 1) were derived from the up curve of the hysteresis loop.

All the suspensions tested exhibit a rheological behaviour of shear-thinning type and a yield stress is also made evident.

As application in process monitoring became feasible when rheological properties are linked to an operative condition, the aim of the present work is to find a mathematical model correlating relative viscosity (*η*_*r*_) to solid concentration TTS% of activated sludge at different shear rates ($\dot{\gamma }$).

The following model of $\eta_{r}=f(\Phi ,\dot{\gamma })$ type was proposed:

(1) $$\begin{array}{c} \eta_{r}={\left\{1-\frac{\left(8-3.5\Phi \right)+\left(8-0.5\Phi \right)\left[\dot{\gamma }/\left(100+100\Phi \right)\right]}{\left(4+0.25\Phi \right)\left[1+\dot{\gamma }/\left(100+100\Phi \right)\right]}\right\}}^{-2}, \end{array}$$

where Φ is TTS%.

Equation (1) was obtained modifying Quemada equation in its adjustable 4 parameters. In detail, one of these (*p*) has been imposed =1, while remaining 3 parameters have been considered Φ linear functions, whose numeric coefficients values has been determined by trial and error fitting every time equation (1) with experimental data by means of simplex method.

Quemada equation finds good application as rheological model of the $\eta_{r}=f(\delta ,\dot{\gamma })$ type in shear-thinning behaviour suspensions and, in particular, if applied to high solid content aqueous suspensions. Its first formulation [17, 18], based on the dissipation energy by viscous friction, can be written as follows:

(2) $$\begin{array}{c} \eta_{r}={\left(1-\frac{k\delta }{2}\right)}^{-2},\\ k=\frac{\left[k_{0}+k_{\infty }{\left(\dot{\gamma }/{\dot{\gamma }}_{\text{c}}\right)}^{p}\right]}{\left[1+{\left(\dot{\gamma }/{\dot{\gamma }}_{\text{c}}\right)}^{p}\right]}, \end{array}$$

where *δ* is the solid volume fraction, *k* is an intrinsic viscosity with limiting values *k*_0_ and *k*_*∞*_ for very low and very high shear rates, respectively, ${\dot{\gamma }}_{\text{c}}$ is a critical shear rate, beyond which the disaggregation process of the disperse phase prevails over the aggregation one, and finally *p* is an empirical exponent, lying within the range 0 to 1. The *p* quantity was tentatively associated with Quemada with particle shape (*p* = 0.5: asymmetric particles; *p* = 1: symmetric particles).

**Figure 3:**
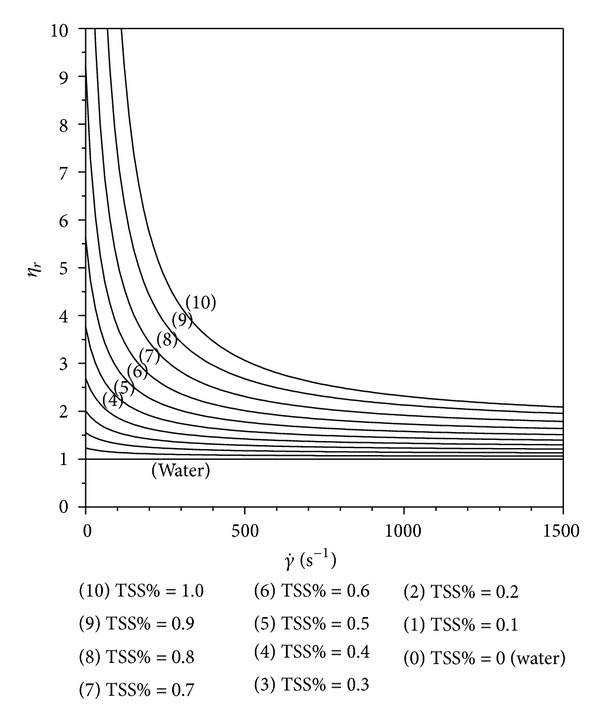
Equation (1) plots at different TSS% of activated sludge.

Equation (1) is drawn in Figure 3 at different TTS% in the 0-1 range. It can be noticed that with TSS% > 1 values the proposed function is not monotonic and so it is unusable having no empirical significance. On the other side TSS% > 1 experimental values were never found.

**Figure 4:**
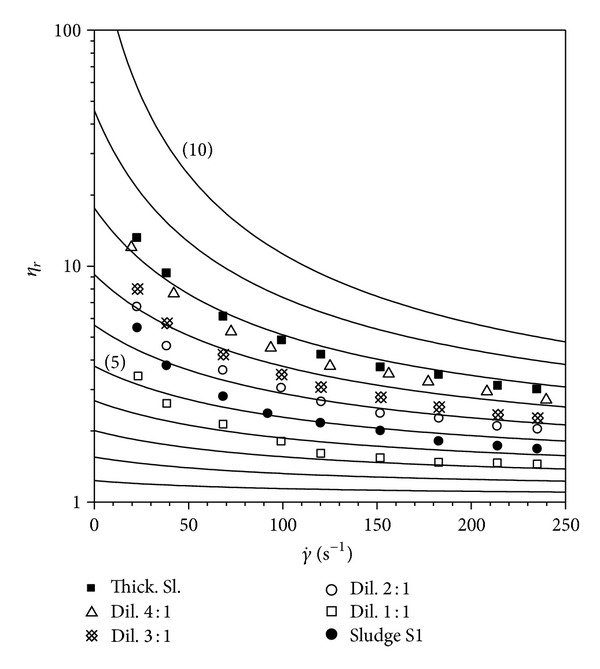
Comparison of equation (1) with experimental flow curves of sludge S1.

**Figure 5:**
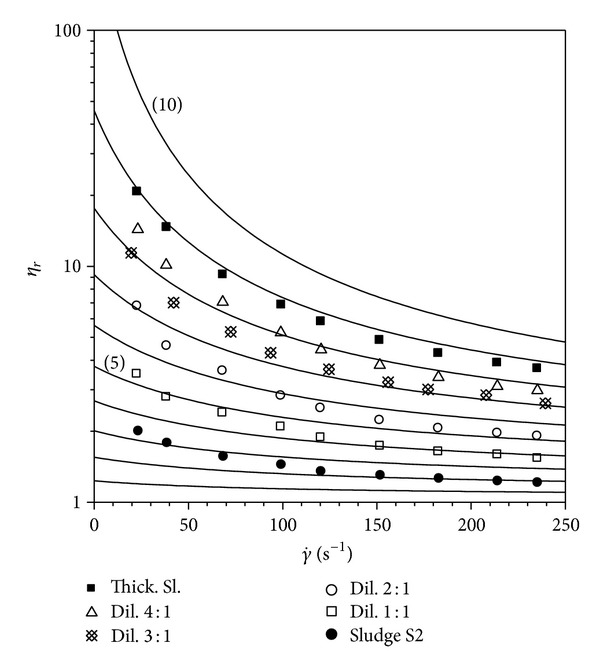
Comparison of equation (1) with flow curves of sludge S2.

**Figure 6:**
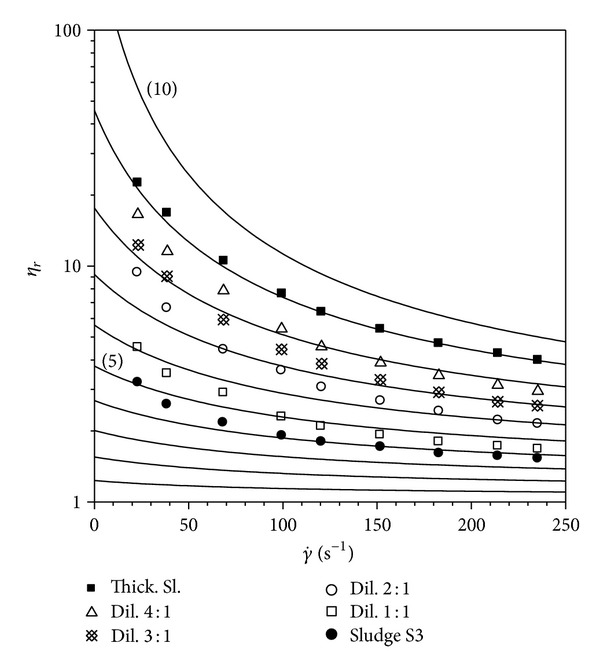
Comparison of equation (1) with flow curves of sludge S3.

A comparison of the model with experimental flow curves of sludge investigated is drawn in Figures 4, 5, and 6.

The fitting of the experimental data with the model is represented in Table 2.

The experimental data detected at a shear rate below 100 s^−1^ were not taken into consideration for the fitting because they are not reproducible in different measurements.

Examination of Table 2 highlights a very good fitting for thickened sludge and a relative error often below 20% between TSS% experimental data and those calculated by the model. Taking into consideration the fact that activated sludge is a low viscous suspension, it often occurs not negligible differences in flow curves obtained repeating twice a rheological measurement of the same sludge. Consequently further two rheological measurements have been accomplished for each sludge reported in Table 2. The further relative error values obtained have not been significantly different to what was described in Table 2 and anyhow an average relative error around 10% is proved for all sludge examined. This means that activated sludge solid concentration could be controlled with a good accuracy in real time by measuring the viscosity of the sludge sampled from the clarifier with a reometer, thus monitoring the proper functioning of the treatment plant.

## 4. Conclusions

Rheological behaviour of recycled sludge from a secondary clarifier of a municipal activated sludge wastewater treatment plant was studied by means of a measuring procedure aiming to realize a standardized method to obtain comparable and reproducible results.

The activated sludge studied exhibits a slightly thixotropic and a shear-thinning rheological behaviour depending on solid concentration. The solid concentration of sludge was correlated to relative viscosity of suspensions by an experimental equation proposed fitting with minor relative error flow curves of activated sludge at different solid concentration. This empirical correlation could be used in monitoring the proper functioning of a treatment plant measuring directly viscosity of sludge.

## Figures and Tables

**Table 1:** Characteristics of tested sludges.

Sample	Initial sludge	Thickened sludge	Dilution 4 : 1	Dilution 3 : 1	Dilution 2 : 1	Dilution 1 : 1
	TSS (g/L)	TSS (g/L)	TSS (g/L)	TSS (g/L)	TSS (g/L)	TSS (g/L)
S1	4.088	8.025	6.420	6.019	5.350	4.013
S2	1.781	8.750	7.000	6.563	5.833	4.375
S3	3.960	8.410	6.728	6.308	5.607	4.205

**Table 2:** Comparison between experimental and calculated TSS% values, relative error, standard deviation (SD) estimates, and coefficient of determination (*R* ^2^) values for the model investigated.

Sludge	TSS (g/L)	TSS% experimen.	TSS% calculated	Relative error	SD	*R* ^2^
S1	4.088	0.41	0.46	0.122	0.094	0.860
Dil. 1 : 1	4.013	0.40	0.30	0.250	0.075	0.900
Dil. 2 : 1	5.350	0.53	0.58	0.094	0.174	0.920
Dil. 3 : 1	6.019	0.60	0.63	0.117	0.139	0.930
Dil. 4 : 1	6.420	0.64	0.74	0.156	0.162	0.946
S1 thick.	8.025	0.80	0.78	0.025	0.095	0.986
S2	1.781	0.18	0.21	0.167	0.037	0.887
Dil. 1 : 1	4.375	0.44	0.41	0.068	0.052	0.907
Dil. 2 : 1	5.833	0.58	0.54	0.069	0.104	0.915
Dil. 3 : 1	6.563	0.65	0.71	0.092	0.172	0.931
Dil. 4 : 1	7.000	0.70	0.77	0.100	0.118	0.975
S2 thick.	8.750	0.87	0.88	0.011	0.252	0.996
S3	3.960	0.40	0.38	0.050	0.084	0.927
Dil. 1 : 1	4.205	0.42	0.45	0.071	0.126	0.917
Dil. 2 : 1	5.607	0.56	0.63	0.125	0.188	0.901
Dil. 3 : 1	6.308	0.63	0.72	0.143	0.234	0.919
Dil. 4 : 1	6.728	0.67	0.78	0.164	0.296	0.950
S3 thick.	8.410	0.83	0.90	0.084	0.495	0.988

## Nomenclature

*k*: Intrinsic viscosity
*k*_o_: Intrinsic viscosity at very low shear rates
*k*_*∞*_: Intrinsic viscosity at very high shear rates
*p*: Exponent in (1)
*R*^2^: Determination Coefficient Value
SD: Standard deviation estimates
TSS: Total Suspended Solids (g/L).

### Greek Letters

Φ: TTS%
$\dot{\gamma }$: Shear rate, s^−1^
$\dot{\gamma_{\text{c}}}$: Critical shear rate, s^−1^
*δ*: Solid volume fraction
*η*_*r*_: Relative viscosity
*η*_*∞*_: Infinite viscosity, mPa s
*τ*: Shear stress, Pa.
